# Where lung cancer and tuberculosis intersect: recent advances

**DOI:** 10.3389/fimmu.2025.1561719

**Published:** 2025-04-02

**Authors:** Chunju Fang, Xuanlu He, Fei Tang, Zi Wang, Cong Pan, Qi Zhang, Jing Wu, Qinglan Wang, Daishun Liu, Yu Zhang

**Affiliations:** ^1^ Department of Oncology, Guizhou Provincial People’s Hospital, Guiyang, China; ^2^ School of Clinical Medicine, Zunyi Medical University, Zunyi, China; ^3^ School of Biological Sciences, Guizhou Education University, Guiyang, China; ^4^ Translational Medicine Research Center, eBond Pharmaceutical Technology Co., Ltd., Chengdu, China; ^5^ Department of Respiratory and Critical Care Medicine, Frontiers Science Center for Disease-related Molecular Network, West China Hospital, Sichuan University, Chengdu, China; ^6^ Department of Respiratory and Critical Care Medicine, Guizhou Provincial People’s Hospital, Guiyang, China; ^7^ National Health Commission Key Laboratory of Pulmonary Immune-Related Diseases, Guizhou Provincial People’s Hospital, Guiyang, China

**Keywords:** lung cancer, tuberculosis (TB), immunosuppressive microenvironments, immunometabolism, inflammation

## Abstract

Lung cancer (LC) and tuberculosis (TB) represent two major global public health issues. Prior evidence has suggested a link between TB infection and an increased risk of LC. As advancements in LC treatment have led to extended survival rates for LC patients, the co-occurrence of TB and LC has grown more prevalent and poses novel clinical challenges. The intricate molecular mechanisms connecting TB and LC are closely intertwined and many issues remain to be addressed. This review focuses on resemblance between the immunosuppression in tumor and granuloma microenvironments, exploring immunometabolism, cell plasticity, inflammatory signaling pathways, microbiomics, and up-to-date information derived from spatial multi-omics between TB and LC. Furthermore, we outline immunization-related molecular mechanisms underlying these two diseases and propose future research directions. By discussing recent advances and potential targets, this review aims to establish a foundation for developing future therapeutic strategies targeting LC with concurrent TB infection.

## 1 Introduction

Globally, about one in five people will develop cancer in their lifetime, with roughly one in nine men and one in twelve women dying from the disease, as per the 2022 data from the International Agency for Research on Cancer (IARC) (1). In 2022, lung cancer (LC) emerged as the most commonly diagnosed cancer, accounting for nearly 2.5 million new cases, which is about 12.4% of all cancers worldwide (1). LC was also the primary cause of cancer-related deaths in 23 countries, with an estimated 1.8 million deaths (18.7%) (1).

Tuberculosis (TB), a contagious disease triggered by the bacterium Mycobacterium tuberculosis (Mtb), significantly contributes to global health issues and ranks among the leading causes of death worldwide. The World Health Organization’s (WHO) Global Tuberculosis Report 2024 estimates that there were an estimated 10.8 million new TB cases worldwide in 2023, resulting in 1.25 million deaths (2). Yet in 2023, TB probably re-emerged as the primary global driver of mortality attributed to a singular infectious pathogen, following 3 years in which it was overtaken by coronavirus disease (COVID-19) (2). TB primarily affects the lungs (pulmonary TB, PTB) but can also impact other body parts.

Emerging evidence reveals a compounding tuberculosis risk profile in LC patients through both disease pathophysiology and therapeutic interventions. Population-level analyses consistently reported that patients with solid cancers face a two to three-fold higher risk of developing active TB compared to individuals without cancer (3–6), with LC patients exhibiting particularly pronounced vulnerability - a meta-analysis of 23 studies quantified this risk at 9-fold higher than the general population (4). This baseline predisposition is amplified by oncologic therapies through distinct immunological mechanisms. Conventional cytotoxic regimens and molecularly targeted agents have been epidemiologically linked to progressive TB risk escalation (7–10). Notably, modern immunotherapies introduce novel immunological susceptibilities: programmed cell death 1/programmed cell death ligand 1 (PD-1/PD-L1) blockade demonstrates dramatic TB reactivation rates (7–17), evidenced by a Singaporean cohort reporting 2.09% incidence post-ICI treatment (17) and a meta-analysis of 27 studies quantifying 2,000 TB cases per 100,000 PD-1/PD-L1 individuals - representing 35-fold population excess risk (18). Moreover, LC patients treated with ICIs had a 6-fold higher rate of developing active TB compared to the general population (19–21). LC itself may increase the risk of TB activation, and the treatment for LC may further elevate this risk. However, more stratified and detailed studies are needed to elucidate this correlation.

Several studies (22–26) increasingly suggest a strong correlation between prior pulmonary TB infection and the development of LC. Furthermore, several recent meta-analyses (27–29) reported a 2–5-fold higher risk of LC for PTB patients. A systematic meta-analysis and review of 29 cohorts and 44 case-control researches (27) indicated a heightened risk of LC for up to two years following a diagnosed TB (HR 5.01; two studies). Moreover, a study (24) discovered that patients with concurrent LC and TB exhibit an 8-fold higher mortality rate compared to those with LC alone. Another study (30) demonstrated that the coexistence of TB and LC increases mortality risk and worsens the prognosis of patients. However, global epidemiological data on their co-occurrence are currently unavailable (31). Therefore, the coexistence of these two diseases warrants significant attention.

The simultaneous occurrence of PTB and LC is becoming more frequent in clinical settings. This emerging comorbidity constitutes a major public health challenge (32), driven by their interrelated epidemiological connections that potentiate mutual disease progression. Such bidirectional intersection significantly complicates clinical diagnosis and treatment approaches. However, little is currently known about immunization-related molecular mechanisms underlying these two diseases. Thus, this review focuses on immunosuppression microenvironments, immunometabolism, cell plasticity, inflammatory signaling pathways, and up-to-date knowledge derived from spatial multi-omics. Such exploration aims to identify viable immunological intervention targets that can offer significant insights for the clinical management of patients suffering from both LC and TB.

## 2 Similarity of molecular mechanisms in TB and LC

### 2.1 Resemblance between the immunosuppression in tumor and granuloma microenvironments

Both cancer and TB involve processes that reflect a coordinated and highly evolved program of immune escape strategies that interfere with innate and adaptive immune response (33). Tumor cells in LC create an immunosuppressive microenvironment, including regulatory T cells (Tregs), myeloid-derived suppressor cells (MDSCs), and exhausted T cells (characterized by impaired immunity) (34–38). On the other hand, TB primarily affects the lungs, triggers a robust immune effect, and inhibits bacterial growth. However, in some cases, the immune system fails to control the infection, leading to granulomas formation, which is a complex process involving immune evasion of the mycobacterium through continued interaction between Mtb-infected macrophages and T cells in response to chronic inflammation (39).

Tregs (a heterogenous sub-group of CD4^+^ T cells) are typically characterized by their role in inhibiting effector cells through mechanisms dependent on direct cell contact or by releasing anti-inflammatory cytokines, including interleukin-10 (IL-10), interleukin-35 (IL-35), and transforming growth factor-beta (TGF-β) (40, 41). Although Tregs were known for their protective role in lessening pathological inflammation in tissues, they also exhibit a suppressive influence on particular anti-tumor and anti-Mtb effects. Various factors, such as increased production of TGF-β or IL-10, and immature/tolerogenic DCs (40), might stimulate the growth of Tregs, leading to negative effector T cell regulation in both the tumor microenvironments (TMEs) (36, 41) and the granuloma microenvironments (GMEs) (42–44).

MDSCs, a diverse group of myeloid cells, proliferate in response to persistent and low-grade inflammation (45). Along with Tregs, they play a role in creating an immunosuppressive setting and blocking T-cell activation in both TB (45) and LC (34, 37). MDSCs produce elevated amounts of molecules that suppress T-cell reactions, such as indoleamine 2,3-dioxygenase (IDO), arginase-1, TGF-β, inducible nitric oxide synthase (iNOS), IL-10, and cyclooxygenase-2 (COX2) (34, 37, 45). PD-L1, IDO, and arginase-1, are crucial in their suppressive functions by breaking down essential metabolites and producing harmful byproducts that accumulate in the TME, thereby hindering T cell proliferation (34, 35, 37, 46). Recent spatial investigations have revealed a consistent structural pattern in TB granulomas, where PD-L1 and IDO1 are spatially synchronized, with myeloid core-infiltrating Tregs and a notable absence of activated T cells (44). Elevated levels of MDSCs have been found in the lungs of patients with TB and LC, suppressing the growth and cytokine release by CD4^+^ and CD8^+^ T cells and regulating T-cell movement (47, 48).

At the core of both the GME and TME is the shared characteristic of exhausted T cells within their local regions. This exhaustion is linked to the decline of robust effector functions, the upregulation of various inhibitory receptors, including immune checkpoints like CTLA-4, TIM-3, LAG-3, and PD-1 (49), and an altered transcriptional program (50). These dysfunctional T cells exhibit various suppressive receptors and/or immune-modulating cytokines, triggering negative regulatory pathways linked to inadequate management of tumors (51, 52), and chronic Mtb infection (53, 54). Moreover, the reduced levels of perforin and granzymes in CD8^+^ T cells has been connected to both Mtb infection (55) and cancer (56), potentially leading to reduced target cell killing.

**Figure 1:**
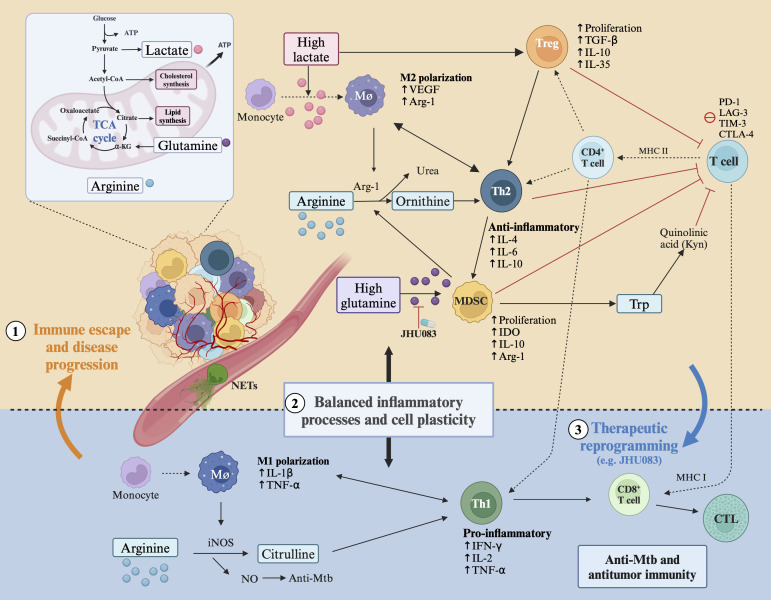
Similarity of molecular mechanisms in TB and LC microenvironment. ① Inflammation-driven metabolic and immune reprogramming, such as lactate, arginine, and glutamine. Expansion and activation of immunosuppressive subsets, including Tregs, MDSCs, and exhausted T cells, leading to immune escape and disease progression. ② Balanced inflammatory processes (Th1 and Th2) and cell plasticity, including immune cell polarization (e.g. M1 and M2 polarization) and spatiotemporal cellular plasticity. ③ This plasticity provides an opportunity for therapeutic reprogramming of the immune system to potentiate anti-Mtb and antitumor activity. For instance, JHU083, a glutamine metabolism antagonist, has been shown to enhance antimycobacterial activity. Abbreviations: Trp, Tryptophan; Kyn, kynurenine; quinolinic acid, a product of the tryptophan/kynurenine pathway; Mø, macrophage; Th, helper T cell; MDSC, myeloid-derived suppressor cell; Treg, regulatory T cell; NETs, neutrophil extracellular traps; NO, nitric oxide; VEGF, vascular endothelial growth factor; IFN-γ, interferon-γ; iNOS, inducible nitric oxide synthase; CTL, cytotoxic T cell; IDO, indoleamine 2,3-dioxygenase; Arg1, L-arginase; IL, interleukin; TGF, transforming growth factor; PD-1, programmed cell death 1; CTLA-4, cytotoxic T-lymphocyte-associated protein 4; LAG-3, lymphocyte activating 3; TIM-3, T cell immunoglobulin and mucin-domain containing-3; TCA cycle, tricarboxylic acid cycle. Created in https://BioRender.com.

In summary, LC and TB weaken the immune system both locally and systemically (**Figure 1**) and highlight the clinical significance of their coexistence. However, the interplay between the similar immunosuppressive microenvironments of tumors and granulomas, particularly the immune subtypes and their differences, requires further investigation. New detection methods, such as spatial multi-omics, are needed to provide insights into these interactions. A recent study (57) demonstrated that the relationship between KRT80 and the progression of lung adenocarcinoma was confirmed through both bulk transcriptome and single-cell transcriptome analyses. However, as the study cohorts were sourced from public databases and the sample size was limited, large-scale cohort studies are needed to fully evaluate the model’s effectiveness. Ideally, sequencing of granuloma and tumor tissue samples from clinical patients with tuberculosis and lung cancer would provide valuable insights. The weakened immune response in TB and LC arises from interactions with specific cells in their surrounding microenvironment. In the following sections, we will systematically compare how these two diseases share common mechanisms in three key areas: persistent inflammatory signals, cellular adaptation mechanisms, and metabolic reprogramming of immune cells.

### 2.2 Inflammation and cell plasticity in TB and LC

Immune system activity is influenced by the proportion of different cell types within specific tissues, the stage of tumor progression or Mtb infection, and the interplay of signaling within the TME or GME, including pro-inflammatory and anti-inflammatory processes (44, 58–60) (**Figure 1**). The characteristics and roles of immune cells are dynamic, changing with their local environment (44, 58–60).

First, this plasticity is evident in diverse cell polarization types, each directed by unique transcriptional programs. For instance, macrophages associated with Mtb infection or tumors originate from tissue-resident cells as well as peripheral blood monocytes are drawn to disease sites as a reaction to inflammatory signals (61–63). Cytokines from the Th1 class, including IFN-γ, encourage the polarization of classically activated M1 macrophages (61, 62). These macrophages, in turn, release pro-inflammatory cytokines such as TNF-α and IL-1β, and exhibit robust antigen processing capabilities, presenting significant antimicrobial and antitumor activities (61, 62). Conversely, Th2 or anti-inflammatory cytokines, such as TGF-β, IL-10, and IL-4, trigger the activation of M2 macrophages, which express arginase-1. These macrophages exhibit weak cytotoxicity, and are associated with wound healing and extracellular matrix remodeling (62, 64, 65).

Macrophages balance pro-inflammatory and anti-inflammatory responses to fight against Mtb infection and tumor cells while controlling tissue pathology. High-dimensional profiling techniques have recently enabled more precise, marker-based definitions of the diverse macrophage populations within tumors and their plastic evolution throughout disease progression or therapy (63, 66). A recent study (67) revealed that patients with PTB exhibit numerous non-necrotizing leukocyte aggregates around necrotizing granulomas. These lesions were more compositionally diverse than the necrotizing type and can be classified into four categories based on the cellular composition and spatial distribution of B cells and macrophages (67). This inherent macrophage plasticity poses a challenge when understanding their complex biology and presents therapeutic targeting opportunities.

Second, spatiotemporal cellular plasticity enables cells to transition between different states during cell migration and metastasis (58). Over the past decade, spatial multi-omics technologies have been employed to study the transcriptomes, proteomes, and metabolomes of the tumor immune microenvironment (TIME) in various cancers (68). For instance, Mark Sorin et al. (38) used highly multiplexed imaging mass cytometry (IMC) to analyze the cellular TIME landscape in lung adenocarcinoma. This approach revealed dynamic cellular behaviors and spatial patterns that were correlated with specific clinical outcomes, including variations in patient survival. Similarly, the polymorphic and elastic nature of TB granulomas is demonstrated by the coexistence of distinct GMEs within an infected individual, each with unique phenotypical and functional properties. Recent studies using MIBI-TOF technology (multiplexed ion beam imaging by time of flight) have identified eight representative microenvironments within TB granulomas (44). These microenvironments are characterized by local immunosuppression markers like PD-L1 and IDO-1 on expanding Tregs, myeloid cells, and elevated levels of TGF-β in the absence of IFN-γ (44). Moreover, the cellular composition of granulomas evolves with the infection stage; early high-burden granulomas (four weeks post-infection) show a type 2 wound healing effect, driven by IL-13 and IL-4, while late low-bacterial-burden granulomas (ten weeks post-infection) are dominated by a type 1 response, characterized by a greater presence of cytotoxic CD8^+^ T cells by pro-inflammatory signaling networks (69). These analyses illustrate that during various stages of tumor development and Mtb infection, cell types exhibit significant plasticity, which is shaped and controlled by factors such as cytokines, inflammation and growth signals, highlighting the importance of spatial relationships in understanding TIME and GME biology.

### 2.3 Immunometabolism in TB that resembles LC

The tumor microenvironment and tuberculous infection are associated with three primary immunity-related metabolic pathways (glucose, fatty acids, and amino acids) and their products (70–72). The three major nutrients and their derivatives produce a broad range of metabolites, which are detected by specific sensors (metabolite sensing). This triggers a series of signal transduction pathways and epigenetic changes that influence gene expression (70). Metabolite sensing in cancer has emerged as a significant area of interest in recent years, but many questions remain unanswered. The regulation of metabolism, immunity, and inflammation via metabolite sensing allows the human body to coordinate its pathophysiology and maintain equilibrium with its external environment (70, 73, 74). Metabolic reprogramming in cancer cells causes them to exhibit different phenotypic characteristics from normal cells, including increased cell proliferation, migration, invasion, and angiogenesis (70, 73, 74). This metabolic disruption in cancer cells further fosters a microenvironment rich in various oncometabolites that promote cancer growth, thereby creating a vicious cycle. Meanwhile, baseline metabolic heterogeneity and inflammation-driven metabolic reprogramming during Mtb infection are associated with crucial immune functions, including mycobacterial growth control and the guidance of protective immunity (71).

The ways metabolites influence these immune cells and aid tumor and TB immune evasion (by impacting the function of immune cells) require further exploration. Given that both the pro- and anti-inflammatory immune cell phenotypes are connected to their cellular metabolism, it is crucial to define the molecular mechanisms that govern their metabolism (71). Arginine, lactate, and glutamine are among the metabolites that display commonality in LC and TB (**Table 1**). This section details how these metabolites and metabolic reprogramming influence immune cells in the tumor and granuloma microenvironments.

**Table 1:** Comparative analysis of immune-metabolic crosstalk in LC and TB microenvironments.

Category	LC	TB	Shared Mechanisms	References
Upstream Pathways	- Warburg Effect (glycolysis) - HIF-1α signaling - Oncogenic mutations (e.g., KRAS)	- Glycolytic reprogramming in early infection - Inflammatory cytokine signaling	Metabolic reprogramming driven by hypoxia/inflammation	(71, 81, 96)
Downstream Effects	- Immunosuppression (Treg/MDSC expansion) - Angiogenesis - Chemoresistance	- Macrophage polarization (M1/M2) - Granuloma formation - Bacterial persistence	Altered T-cell function, macrophage plasticity	(76–78, 82)
Arginine Dynamics	- Arg-1 activity depletes arginine → T-cell suppression - M2 polarization	- iNOS-driven NO production (M1) - Arg-1 promotes tissue repair (M2)	Arg-1/iNOS balance regulates immune cell function	(46, 79, 80)
Lactate Role	- Lactate induces M2 polarization, PD-1 signaling - Promotes chemoresistance	- Lactate accumulation in granulomas → T-cell dysfunction - Links to chronicity	Lactate drives immunosuppressive microenvironment	(81–96)
Glutamine Dependency	- Fuels tumor growth - MDSCs and Tregs recruitment - Suppresses effector T-cells	- Elevated glutamine recruits MDSCs - Suppresses effector T-cells	Glutamine supports immunosuppressive cell populations	(97–100)

#### 2.3.1 Arginine dynamics in macrophage polarization and T-Cell function

Classically activated M1 macrophages transform arginine into NO via iNOS activity, while M2 macrophages metabolize arginine to ornithine and urea by arginase-1 (64, 75). Pro-inflammatory glycolytic M1 macrophages, known for their antimicrobial activities, can transition to anti-inflammatory M2 macrophages, which depend on oxidative phosphorylation metabolism and lack antimicrobial effect (71, 76–78). This transition helps regulate inflammation and fosters tissue repair throughout the chronic stage of Mtb infection (71, 76–78). Increasing L-arginine levels have also been demonstrated to trigger metabolic alterations, including a transition from glycolysis to oxidative phosphorylation in activated T cells, promoting the formation of central memory-like cells with enhanced survival capacity and anti-tumor activity (46). Arginase-1 breaks down arginine into ornithine and contributes to the regulation of immune function in activated T-cells by depleting arginine in the local microenvironment (controlling arginine bioavailability results in nitric oxide production control), as demonstrated in cancer and tuberculosis experimental models (46, 79, 80). Overall, arginine influences macrophage polarization and affects T-cell function in both TB and LC. However, the specific impact of arginine on the immune microenvironment and systemic immune status in patients with concurrent TB-LC needs further investigation.

#### 2.3.2 Lactate’s role in the tumor and granuloma microenvironments

In solid tumors, lactate buildup occurs in the TME under hypoxic conditions and even in the presence of oxygen, driven by the Warburg Effect. This shift toward increased glycolytic flux is closely correlated with malignant transformation (81). Lactic acid has been linked to histone regulation and epigenetic lactylation in macrophage genomes (82–84). It promotes the transformation of macrophages from the pro-inflammatory, anti-cancer M1 to the anti-inflammatory, pro-cancer M2 type (82–84). Lactic acid can also enhance the immune suppressive function of Tregs (85), be converted to acetic acid by tumor-associated fibroblasts for cancer cell utilization (86), and subvert PD-1 inhibitor function to promote immune suppression (87). A recent study demonstrated that alanyl-tRNA synthetase (AARS1), which functions as a lactate sensor and lactyltransferase, can lactylate p53 and contribute to tumorigenesis (88). Additionally, lactate-induced lactylation of NBS1 was reported by Chen et al. (89) to promote homologous recombination (HR)-mediated DNA repair and lead to chemotherapy resistance.

Macrophages shift toward glycolytic metabolism in the early stages of Mtb infection, increasing lactate production (90, 91). Granuloma lesions can exhibit high lactate concentrations, reaching levels comparable to those observed in tumors (92, 93). Mtb expresses cytochrome bd oxidase to oxidize lactate into pyruvate, fueling bacterial respiration in human macrophages (94). This lactate oxidation capacity correlates with enhanced intracellular growth rates under hypoxia, suggesting lactate serves dual roles as both an immunosuppressive modulator and a pathogen nutrient source. The regulation of T-cell balance is crucial for either exacerbating or resolving TB pathology. The buildup of lactate within the granuloma lesions may significantly influence this balance, ultimately determining whether an anti-inflammatory or pro-inflammatory microenvironment develops (95). Therefore, it is promising to consider whether research approaches related to lactate in the tumor field can be applied to tuberculosis, particularly in exploring the mechanisms of drug-resistant tuberculosis.

#### 2.3.3 Glutamine metabolism and immunosuppression

High glutamine metabolism in tumors was discovered to have a strong correlation with the increased presence of immunosuppressive cells such as MDSCs and Tregs and concomitant suppression of effector T cells, which contributes to the spread of cancer and tumor development (70, 96, 97). Similarly, several studies have indicated that Mtb lung infection creates localized microenvironments characterized by increased elevated glutamine levels (98–100), subsequently leading to the accumulation of MDSCs, diminished effector T-cell activity, and reduced production of NO and citrulline (98). Treatment with JHU083, a glutamine metabolism antagonist, was linked with increased citrulline and NO production levels and potentiated antimycobacterial activity (98). Given the accumulation of glutamine in the tissue microenvironment and its association with immunosuppression, glutamine can be considered a potent target for immunometabolism in TB and LC.

To summarize, the function and persistence of immune cells in TB and cancer can be influenced by metabolic pathways through alterations in inflammatory processes within the tissue microenvironment (101) (**Figure 1**). Dynamic alterations in intracellular and extracellular metabolites, particularly variations in nutrient concentrations, can influence cell signaling pathways and epigenetic gene expression by acting as metabolite sensors (70). This creates a favorable microenvironment for tumor progression (70) or Mtb infection (90). It is important to emphasize that individual metabolic processes have been involved in eliciting effects during Mtb infection and LC progression. However, the inherent interconnectedness of metabolic reactions presents a significant challenge for researchers in identifying the specific contributions of these and other pathways to Mtb infection and LC progression, especially regarding the immune reprogramming related to metabolic pathways and host immune suppression. Additionally, metabolomics is closely intertwined with microbiomics. Notably, recent study (102) on the lung microbiome underscores its critical role in *respiratory* disease mechanisms, suggesting that spatial multi-omics analyses integrating metabolomics and microbiomics represent a promising direction for future research to further explore the molecular mechanisms linking TB and LC.

LC and TB show similarities in their shared immunosuppressive mechanisms, dysregulated inflammatory signaling, cellular plasticity, and immunometabolic reprogramming. These overlapping features may partially explain the pathophysiological basis for their clinical co-occurrence. However, it is important to note that distinct molecular mechanisms exist between the immune microenvironments of TB and LC. While a comprehensive comparative analysis of these nuanced differences is beyond the scope of this review due to space constraints, their exploration remains a critical frontier for future research—a primary motivation for this review. The following section will emphasis the specific molecular pathways, underlying their bidirectional pathological interactions.

## 3 Immunization-related molecular mechanisms in LC→TB

### 3.1 TB activation following immune checkpoint inhibitors

Clinical case observations have shown that enhancing T-cell immunity in cancer patients by blocking the PD-1/PD-L1 axis may lead to latent tuberculosis reactivation (7–21). Studies in mice have demonstrated that PD-1-deficient mice experience intense inflammation and rapid mortality following Mtb infection (9, 103, 104). Additionally, in rhesus macaques, PD-1 inhibition results in larger TB granulomas (105). Several mechanisms may elucidate why ICIs are effective in treating lung cancer but also lead to TB re-activation.

The immune checkpoints balance pro-inflammatory and anti-inflammatory effects to control infections while avoiding immunopathology. In patients infected with Mtb, immune cells like CD4^+^ T cells, NK cells, neutrophils, and monocytes exhibit elevated PD-1 (106–108). In TB patients, PD-1 is present in granuloma areas where the interaction between host and pathogen is balanced but is missing in areas with caseating granulomas and marked immunopathology (107). Inhibiting PD-1 may disrupt the anti-Mtb-specific T-cell balance. This imbalance results in a pro-inflammatory environment rich in TNF-α, IL-6, and IL-12, which can promote Mtb growth, particularly TNF-α (107). It has been demonstrated that TNF-α can trigger necrosis in macrophages infected with Mtb. ROS production is essential for this effective immune response to TB (109–111). However, excessive TNF-α may impair macrophage function (109, 110). By enhancing the immune system’s response, immune checkpoint inhibition may cause inflammatory side activities, commonly known as immune-related adverse events (irAEs), which are autoimmune in nature (112). It has been found that autoreactive T-cells increase in TB patients, suggesting that autoimmunity maybe significant in TB pathology (113). Notably, irAEs caused by ICIs can be treated with anti-TNF-α antibodies (114), indicating that TNF-α may be a key factor in autoimmunity and TB pathology following PD-1 inhibition (107).

Moreover, Mtb may possess pathogen-specific characteristics that enable it to thrive in a hyper-inflammatory environment (such as those induced by ICIs) (115). Enhanced T-helper (Th) type 1 (Th1) responses triggered by ICIs can lead to overproduction of cytokines and increased MMP expression, resulting in tissue damage and destabilizing the delicate host-pathogen balance in the lungs. It has been suggested that various inhibitory receptors play crucial roles in modulating TB immune control. For instance, mice deficient in the chemokine scavenger D6 or the negative regulator of the IL-1 system (Toll/IL-1 receptor 8; TIR8) succumbed quickly to low-dose Mtb challenges, experiencing considerable local and systemic inflammation (116, 117). Heightened inflammation can lead to several detrimental outcomes, including an over-recruitment of inflammatory cells and the breakdown of the extracellular matrix, which promote Mtb proliferation (118–120). In this context, blocking PD-1 can induce T-cell dysfunction marked by hyperactive inflammatory responses, leading to a loss of immune regulation (121, 122).

In summary, the emerging clinical findings, together with studies in animal models and advanced cell cultures, collectively indicate that PD-1/PD-L1 disruption may create hyperinflammatory environments that favor mycobacterial growth. However, immune checkpoint inhibition does not necessarily result in autoimmune disorders or excessive inflammation; it depends on whether the balance is disrupted. Immune checkpoint inhibitors are widely used in cancer therapy, but their potential application in the treatment of tuberculosis remains controversial and needs further investigation (123). The key is to identify which patient groups are at higher risk for tuberculosis reactivation when receiving immune checkpoint inhibitors, thereby providing guidance for clinical practice.

### 3.2 TB activation following surgery, chemotherapy, and/or radiotherapy

Epidemiological studies found that anti-tumor therapy (surgery, chemotherapy, or radiotherapy) further increased the risk of Mtb infection in cancer patients (20, 21). Recent case reports have indicated that triple cancer therapy -which combines immunotherapy and chemoradiotherapy- may be linked to an increased risk of TB reactivation (124, 125). Lung cancer itself may increase the risk of tuberculosis activation, and the treatment for lung cancer may further elevate this risk. However, more stratified and detailed studies are needed to elucidate this correlation.

The induction of lymphopenia in cancer patients treated with most chemotherapeutic drugs has been acknowledged since the 1980s (126). Radiotherapy can cause lymphopenia as a direct result of blood or bone marrow irradiation (127–129). Additionally, 30% of patients undergoing anti-EGFR therapy develop lymphopenia (130). Multi-tyrosine kinase inhibitors can also induce grade III/IV lymphopenia in 10–15% of patients (131). As the combination of immunotherapy with chemotherapy, chemoradiotherapy, or targeted therapy becomes increasingly common in lung cancer treatment (132–135), combined treatment-induced lymphopenia warrants greater attention (128, 129).

Lymphocytes are susceptible to radiotherapy, and a reduction in peripheral total lymphocyte count (TLC) following ionizing radiation indicates an impaired host immune response to cancer. Numerous studies have established a connection between radiation-induced lymphopenia and poor prognosis across various cancers, including lung cancer (127–129, 136). One study involving 268 patients with advanced NSCLC who received ICIs, with 146 of them also undergoing radiotherapy, found that patients with lymphopenia at the start of immunotherapy had markedly poorer progression-free survival (PFS) (2.2 vs. 5.9 months) and overall survival (OS) (5.7 vs. 12.1 months) (136). Another recent study (129) reported that the median TLC dropped from 1.52 × 10^9 cells/L to 0.72 × 10^9 cells/L after chemoradiation in patients with locally advanced NSCLC, with 23% developing severe lymphopenia (<0.5 × 10^9 cells/L). Severe lymphopenia presented at the start of immunotherapy continued to be an independent negative predictor of PFS, with an HR of 4.90 and p < 0.001. Additionally, higher TLCs have been associated with a higher response rate and more durable treatment effects in patients treated with ICIs (137–139). In recent years, stereotactic body radiation therapy (SBRT) for lung cancer has garnered significant attention. Compared to conventional fractionated radiotherapy, SBRT can reduce lymphocyte depletion, improve prognosis, alleviate immunosuppression, and synergistically enhance the efficacy of ICIs, thereby boosting antitumor effects (140).

**Figure 2:**
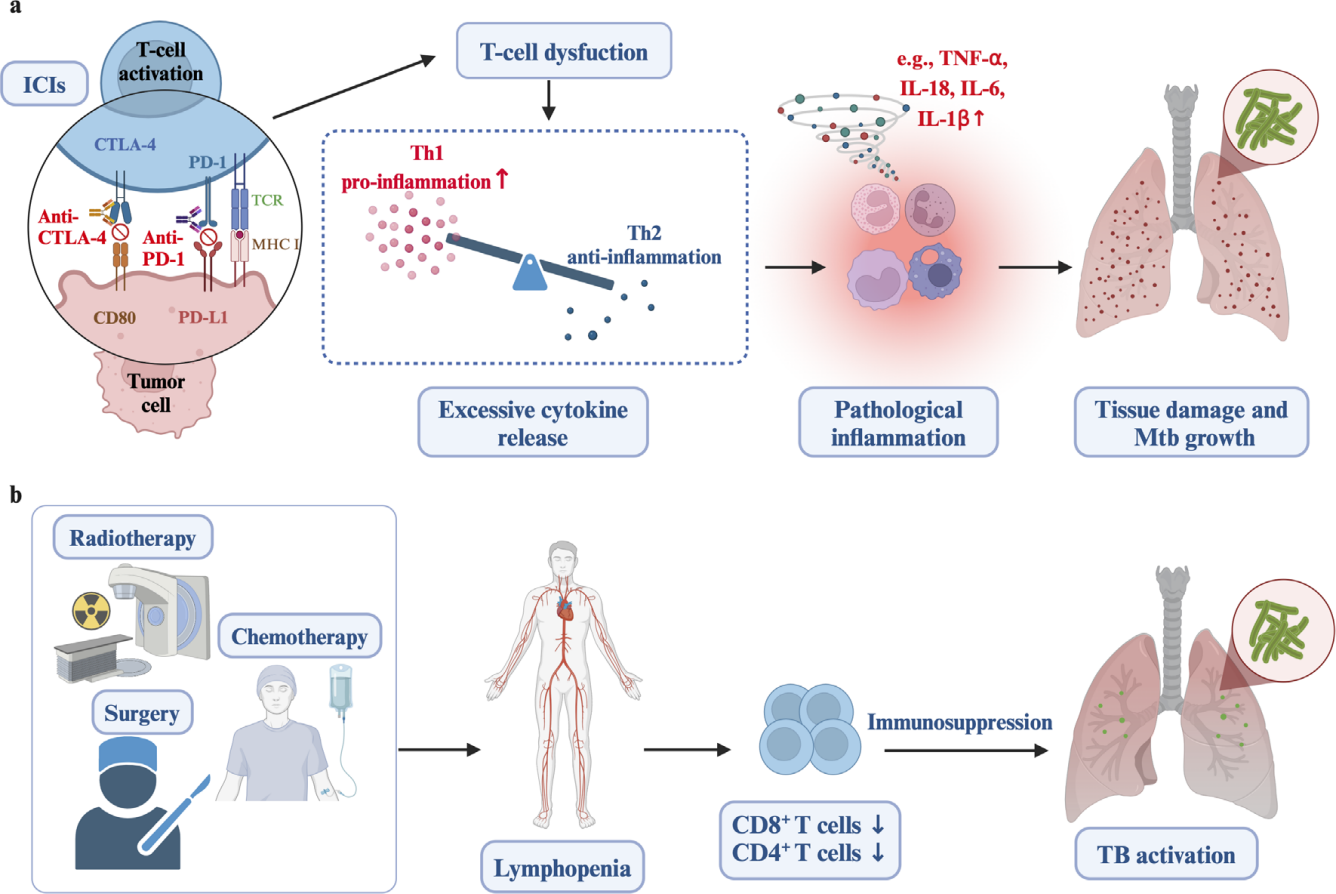
The relationship between cancer treatments and increased risk of active tuberculosis. **(a)** Immune checkpoint inhibitors (ICIs) can cause T-cell dysfunction and hyperinflammatory conditions, including excessive TNF-α, IL-6, and IL-12, resulting in tissue damage and mycobacterial growth. **(b)** Treatments such as radiotherapy, surgery, and chemotherapy lead to lymphopenia, decreased CD8^+^ T cells, systemic immunosuppression, and reduced infection barriers, ultimately increasing the susceptibility to active tuberculosis. Created in https://BioRender.com.

To conclude, a primary commonality between TB and LC is immunosuppression. The immunosuppression resulting from LC itself could potentially heighten the risk of TB activation. Furthermore, cancer treatments, such as immunotherapy, surgery, chemotherapy, and radiotherapy, might cause myelosuppression and immune dysregulation, thereby elevating the risk of active tuberculosis (**Figure 2**). While this statement is a hypothesis, and the detailed immunization-related molecular mechanisms involved need to be further explored. In clinical practice, we need to be aware of the immunosuppression that occurs during anti-tumor treatment, particularly the reduction in lymphocyte counts, and disruptions in immune function. Additionally, high-risk populations for tuberculosis activation should be screened and monitored before and during lung cancer treatment.

## 4 Immunization-related molecular mechanisms in TB→LC

### 4.1 TB contributes to tumor initiation and promotion

Successful tumor initiation requires two main interconnected events. 1) The buildup of mutations or epigenetic changes in genes and cellular signaling networks involved in tumor suppression (inactivation) and the activation of oncogenic signals is crucial (58). Inflammatory effects possess potent mechanisms that contribute to the accumulation of mutations and epigenetic changes in nearby epithelial cells, while these have traditionally been associated with environmental factors (such as variable radiation, carcinogens, and/or UV), and errors in DNA replication and repair. The initiation phase of tumorigenesis often involves irreversible DNA changes induced by reactive nitrogen species (RNS) and reactive oxygen species (ROS). These reactive species are mainly produced by macrophages and neutrophils, which is involved in inflammatory response derived from Mtb infection (58, 141–144). 2) For transformed or malignant clones to progress into a fully developed tumor, their emergence must be accompanied by substantial growth, a process significantly driven by inflammatory mechanisms (58).

**Figure 3:**
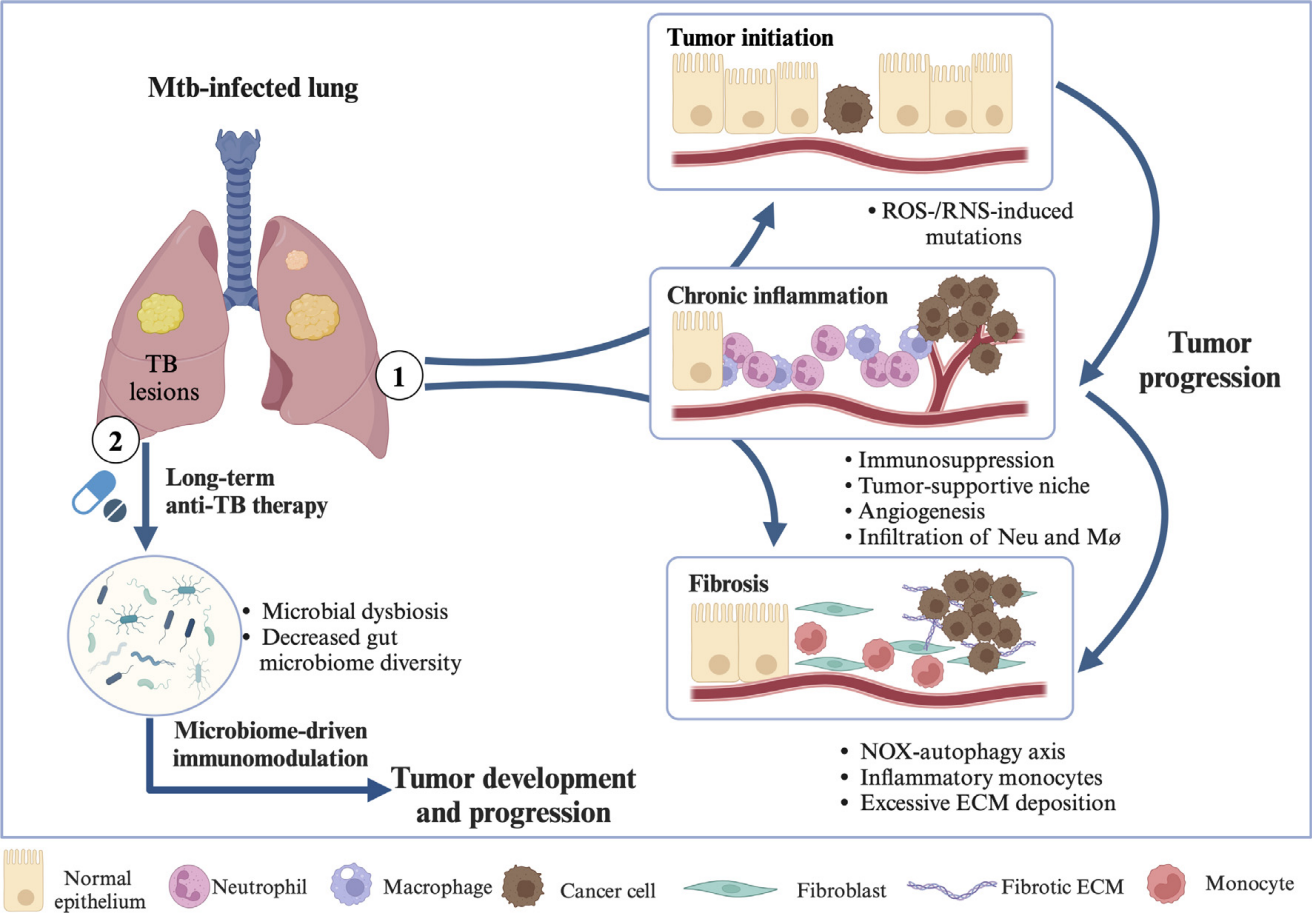
Molecular mechanisms in TB→LC. ① Chronic inflammation driven by Mtb may contribute to LC initiation, promotion, progression, and metastasis through mechanisms such as ROS and RNS production, DNA damage and repair, exhausted T cell phenotypes, induced immunosuppression, angiogenesis, tuberculous fibrosis, NOX-autophagy axis, excessive ECM deposition, and the infiltration of neutrophils, macrophages, and inflammatory monocytes. ② Microbial dysbiosis and reduced gut microbiome diversity, resulting from prolonged antibiotic treatment for tuberculosis, may be associated with the development and progression of LC through microbiome-driven immunomodulation. Abbreviations: ROS, reactive oxygen species; RNS, reactive nitrogen species; ECM, extracellular matrix; Neu, neutrophil; Mø, macrophage; NOX, non-phagocytic cell oxidase. Created in https://BioRender.com.

The ‘initiation’ phase involves permanent DNA alterations that can remain in normal cells until the ‘promotion’ stage is triggered. The ‘promotion’ phase is crucial as it facilitates cell proliferation, ROS and RNS production, recruitment of inflammatory cells, and oxidative DNA damage (58, 59). Inflammatory cytokines such as IL-6, IL-17, and IL-11 play a vital role in shaping cell plasticity within the TME and enhancing tumor cell proliferation, particularly under sub-optimal *in vivo* conditions such as hypoxia, nutrient deficiency, and limited production of growth factors, while also counteracting anti-tumor immunity (58, 145). Moreover, inflammatory signals modulate the structural and metabolic properties of tumor and stromal cells by modulating the extracellular matrix, growth factor availability, and key metabolites linked to redox and amino acid metabolism (145). Prolonged pathogen-induced chronic inflammation significantly elevates the risk of cancer (58). Chronic inflammation triggered by TB infection induces DNA damage, DNA repair, heightened production of ROS and RNS, cell death, as well as cell proliferation (146). These processes remain in chronic inflammatory tissues, leading to uncontrolled DNA replication and cell proliferation, possibly contributing to LC initiation and promotion (**Figure 3**).

### 4.2 TB promotes tumor progression and metastasis

As previously discussed, the formation of granulomas is a complex process that involves Mtb immune evasion through ongoing interactions between infected macrophages and T cells in response to chronic inflammation (39). The TME and TB granulomas exhibit a central theme of exhausted T cell phenotypes (33). Numerous immune checkpoints, such as CTLA-4, TIM-3, LAG-3, and PD-1, are enriched in these exhausted T cells and are crucial in inhibiting T cell effects (49). The development of tumors may be influenced by the exhausted phenotype of T cells and the immunosuppressive effects triggered by Mtb infection.

Furthermore, neutrophils are important in the prolonged pulmonary inflammation associated with TB. As innate immune cells, neutrophils are involved in the early phases of Mtb infection and crucial for killing the bacteria (147). Beyond their phagocytic and lytic roles, neutrophils undergo necrosis and release neutrophil extracellular traps (NETs), which act to capture and sequester extracellular bacteria, thereby restricting their dissemination (148). However, during later phases of infection, uncontrolled neutrophil activity leads to neutrophil-driven inflammation, exacerbating lung tissue damage (147, 149–152). Studies show that NET formation can promote Mtb growth and contribute to disease severity and inflammation-mediated tissue damage (147, 149, 150). The inflammatory response is primarily driven by three neutrophil functions: oxidative burst, necrosis, and NETosis (153). The buildup of neutrophils at inflammation sites results in uncontrolled necrosis and inflammation in the surrounding tissues. Similarly, NETs are detected in necrotic lung lesions of TB patients who show poor responses to antibiotic therapy, highlighting their involvement in the pathogenesis of late-stage TB (141, 150, 154, 155).

Meanwhile, cancer cells can manipulate neutrophil recruitment and behavior by converting some into a pro-tumor phenotype (156, 157). Pro-tumor neutrophils can interact with cancer cells and other TME cells, including T cells, macrophages, and stromal cells, to promote tumor initiation, growth, and metastasis (156–159). This indicates that neutrophil-driven inflammation, characterized by the buildup of proinflammatory proteins linked to neutrophils and the formation of NETs, which work together to harm lung tissue in TB may contribute to LC progression and metastasis. However, the precise molecular mechanisms underlying this process require further investigation.

Repeated tissue damage and repair, caused by prolonged and intense TB pulmonary inflammation, ultimately lead to fibrotic scarring that is linked to a heightened risk of lung cancer (160–162). Recent studies in mouse models have documented that tuberculous fibrosis can elevate the tumorigenic potential of lung cells, potentially mediated by NOX4-associated autophagy (163). Inflammatory monocytes (IMs), identified as the “CCR2^high^CD14^+^ CD16^low^” phenotype in humans, are present in lung cancer and critical in forming scar tissue and promoting tumors (164–167). A recent study demonstrated that CCR2^+^ monocytes in the lungs of mice with cystic fibrosis drive pathogenic TGF-β signaling and maintain a pro-inflammatory environment by facilitating neutrophil recruitment (167). Lung squamous cell carcinomas (SCC) are marked by poor survival outcomes and significant infiltration of IMs, creating conditions that facilitate the distant metastasis of SCC (166). Tumors attract IMs by secreting monocyte chemoattractant protein-1 (MCP-1 or CCL2) (166), which can promote tumor cell proliferation, migration, and survival (168). High levels of CCL2 expression and increased macrophage infiltration have been linked to a poor prognosis and the development of metastatic disease in cancer (169). Additionally, elevated levels of CCL2 are also found in patients with pulmonary tuberculosis (170).

In summary, inflammatory signals can modify the TME to promote immunosuppression through the actions of Tregs, immature myeloid cells, and other suppressive elements. These signals also enhance the recruitment and proliferation of various pro-tumorigenic auxiliary cells within the TME while promoting their specialized functions (58). Chronic inflammation driven by Mtb may contribute to the progression and metastasis of lung cancer through mechanisms such as ROS and RNS production, DNA damage and repair, exhausted T cell phenotypes, induced immunosuppression, tuberculous fibrosis, and the infiltration of neutrophils and inflammatory monocytes (IMs) (**Figure 3**).

### 4.3 Risk factors for LC induced by anti-TB therapy

Advancements in sequencing technologies and analytical methods have enabled researchers to demonstrate that the gut microbiota performs various functions that impact the host’s overall health, such as nutrient metabolism and the regulation of the immune system by influencing immunological, inflammatory, neurological, metabolic, and endocrine functions (171–173). Studies have indicated that certain gut bacterial groups, like Bacteroides and Ruminococcus, in the microbiota of lung cancer patients are linked with early-stage lung cancer (174). Recent research has revealed that reduced gut (and lung) microbiota diversity, the abundance of specific microbiota, and enriched metabolic pathways may be related to lung adenocarcinoma development/progression (175, 176). A study analyzing lung cancer tissues found that bacterial genera such as Romboutsia, Novosphingobium, and Acinetobacter were more abundant than in adjacent normal tissues (177).

Broad-spectrum antibiotic treatment has been shown to heighten susceptibility to Mtb infection and modulate pulmonary inflammatory responses in a murine model (178). Analysis using random forest regression on microbiome-transcriptome-sputum data indicates that normalizing the TB inflammatory state is linked to the clearance of Mtb pathogens, a higher abundance of Clostridia Clusters IV and XIVa, a reduced presence of Bacilli and Proteobacteria (179). Mtb pathogen clearance is associated with the renormalization of the TB inflammatory state. This process is linked to an increase in Clostridia Clusters IV and XIVa and a decrease in Bacilli and Proteobacteria. Additionally, the alterations in inflammatory gene expression accompanying TB treatment are linked to the antimicrobial effects of the drugs, resulting in pathogen clearance and antibiotic-induced shifts in microbiome composition (179).

Moreover, another study demonstrated that gut microbiome dynamics and TB clearance are predictive cofactors in resolving TB-driven inflammation, thereby guiding therapeutic strategies for multi-drug-resistant tuberculosis (180). Antibiotic-induced reductions in pathogen load and microbiome alterations induced by antibiotics are independently linked to changes in inflammatory responses to the treatment of active TB (179). Microbial dysbiosis and decreased gut microbiome diversity (induced by long-term antibiotic treatment against TB) may be related to the occurrence and progression of lung cancer through microbiome-driven immunomodulation (**Figure 3**). However, more detailed research is needed to confirm this relationship. It is also important to explore which specific microbes are involved and play a key role. Additionally, investigating whether probiotics can help modulate the immune system, reduce drug-resistant tuberculosis, and lower the risk of cancer development is a promising area for future research.

## 5 Treatment strategies and future perspective

### 5.1 Discriminating TB and LC: emerging techniques

The concurrent LC and PTB pose substantial difficulties for differential diagnosis. The primary diagnosis methods rely on clinical symptoms, distinctive imaging findings, and pathogen detection. While clinical symptoms may provide indications, they often lack high specificity. Imaging signs reveal that patients with coexisting PTB and LC demonstrate a higher frequency of pleural indentations, satellite lesions, masses, spicule features, small vacuole features, nodular shadows, vacuole features, and lobulation features than those with PTB alone (181). Lung biopsy pathology remains the definitive standard for distinguishing LC from PTB, but acquiring biopsy samples from certain puncture-unfriendly locations can be problematic.

As an alternative, blood-based pathogen detection has emerged. The Hu laboratory (182) has pioneered an ultrasensitive CRISPR-mediated tuberculosis (CRISPR-TB) assay for detecting Mtb-cfDNA in serum, showing potential for improving the identification of pediatric tuberculosis and HIV-associated tuberculosis and allowing for early diagnosis and swift monitoring of tuberculosis treatment responses. Moreover, they have employed a nanoparticle-enhanced immunoassay for the multiplex detection of two interacting Mtb biomarkers on the surface of circulating extracellular vesicles, enhancing tuberculosis diagnosis in immunosuppressed children with HIV (183). Recently, circulating tumor DNA (ctDNA) has surfaced as a minimally invasive biomarker for tumor molecular profiling. The European Society of Medical Oncology (ESMO) has endorsed ctDNA testing in routine clinical practice for tumor genotyping, guiding molecularly targeted therapies in patients with metastatic cancer (184, 185).

With the advent of these non-invasive, highly sensitive technologies for detecting free DNA, the differentiation between PTB and LC is set to become more refined, paving the way for subsequent TB and LC treatments. In PTB-LC differential diagnosis, this CRISPR-TB technology provides two critical functions: 1) Active TB exclusion: A negative CRISPR-TB result (Mtb-cfDNA <5 copies/mL) in patients with lung masses strongly suggests LC-driven pathology, prompting immediate oncological intervention. 2) Treatment response monitoring: In confirmed PTB-LC cases, longitudinal Mtb-cfDNA quantitation (e.g., 80% reduction after 2-month HRZE therapy) helps distinguish TB recurrence from cancer progression when radiographic abnormalities persist. While CRISPR-TB and ctDNA were initially developed for standalone applications, their integration creates a synergistic diagnostic paradigm. The CRISPR-TB assay’s capacity to quantify Mtb-cfDNA load complements ctDNA’s ability to track tumor evolution, enabling real-time differentiation between TB-driven inflammation and LC progression. This dual-liquid biopsy approach may redefine clinical guidelines for TB-LC co-diagnosis in high-burden settings. However, its high cost currently limits widespread adoption.

### 5.2 Advancements in therapeutic targets

#### 5.2.1 Strategies for modulating inflammatory signaling pathways

Pulmonary complications such as fibrosis and cavitation, arise from persistent inflammation driven by host pro-inflammatory signals triggered by a substantial Mtb burden. The effort to shorten treatment durations and enhance clinical outcomes has prompted a two-fold strategy: developing new antimicrobials and host-directed therapies (HDT) that positively influence immune responses to Mtb (147). Targets for HDT in PTB include lipid mediators, matrix metalloproteinases, cytokines, and NETs (147). HDT strategies focus on modulating the immune system by boosting antimicrobial response while suppressing excessive or unhelpful inflammatory effects, thereby reducing lung damage and hastening symptom resolution (147).yole of inflammation as a link between LC and PTB was discussed in Section 2.2; this section will highlight the progress in targeting inflammatory signaling pathways with nonsteroidal anti-inflammatory drugs (NSAIDs).

NSAIDs, widely used to manage various chronic conditions, function as anti-inflammatory medications by inhibiting cyclooxygenase enzymes. Maintaining a balance between LXA-4 and PGE-2 plays a vital role in controlling inflammation derived by TB. The promotion of LXA-4 generation and the reduction of PGE-2 production by NSAIDs contribute to a decrease in TNF levels, ultimately resulting in reduced tissue inflammation (186, 187). A randomized phase II clinical trial revealed that aspirin has been employed as an adjuvant to standard anti-TB therapy for tuberculous meningitis, demonstrating a reduction in stroke risk and mortality rates (188). In a C3HeB/FeJ mouse model of Mtb infection, ibuprofen significantly decreased neutrophil infiltration and reduced pathological lung inflammation in mice (189). Recently, another phase IIb randomized control trial (NCT04575519) has been registered to evaluate the potential benefits of adding acetylsalicylic and acid ibuprofen to the standard TB therapy protocol, targeting both drug-sensitive and multidrug-resistant TB cases.

In line with inflammation’s role in promoting tumorigenesis, long-term NSAID use is linked to reduced cancer incidence (190), including a significant decrease in lung cancer incidence among chronic smokers (191). Furthermore, a phase III trial observed a notable reduction in LC incidence while investigating a blocking antibody aimed at the inflammatory mediator IL-1β for atherosclerosis (CANTOS; NCT01327846) (192). It is worth noting that not every chronic inflammatory disease is associated with an increased cancer risk. Rheumatoid arthritis and psoriasis, for instance, do not seem to contribute to cancer development. However, the use of anti-inflammatory drugs to manage these inflammatory conditions, including TB, might influence cancer risk. Therefore, targeting inflammatory pathways might reduce the severity of both TB and LC, offering a potentially effective approach to enhance long-term outcomes in patients affected by these conditions. Nevertheless, future challenges lie in validating the dose-response relationships of repurposed drugs (e.g., NSAIDs).

#### 5.2.2 Modulation of immunometabolism pathways: novel approaches

The previously discussed roles of immunometabolism in TB and LC highlight their similarities. As an emerging area of research, therapeutic approaches focusing on immunometabolism pathways have seen notable advancements. For example, the glutamine metabolism pathway could be a therapeutic target for tumors and TB. On one side, glutamate’s biological impacts can be lessened by blocking its receptors, including genetic alterations (193) or the use of metabolic glutamate receptor 5 antagonists (194). On the other side, direct inhibition of tumor growth can be achieved by blocking glutamine transport (195, 196), including using the recombinant solute carrier family 7, member 11 (SLC7A11 or xCT). Glutamic acid (a byproduct of glutamine hydrolysis) is able to be expelled from tumor cells via transporters like xCT. The disruption of the glutamate-cysteine anti-transporter xCT results in cellular glutamate retention, causing glutamate to lose its function outside the cells, leading to decreased NSCLC proliferation and invasion (197).

Similarly, several studies have indicated that Mtb lung infection creates localized microenvironments characterized by increased glutamine levels (98–100), subsequently leading to MDSC accumulation, diminished effector T-cell functionality, and a decrease in the synthesis of NO and citrulline (98). Parveen et al. (98) demonstrated that JHU083, a glutamine metabolism antagonist, could trigger earlier recruitment of T-cells, boost infiltration of proinflammatory myeloid cells, and decrease IL-10-producing MDSCs. Inhibiting glutamine metabolism with JHU083 provides both antibacterial and host-targeted actions against tuberculosis (98), offering new perspectives for treating drug-resistant TB. However, its penetration capability into human granuloma microenvironments remains to be validated.

Metabolic disturbances in the tumor microenvironment result in spatial gradients arising from varying lactate sensitivity among tumor cells and stromal cells, including tumor-associated macrophages. Limited perfusion plays a role in creating metabolic gradients, enabling cancer cells to endure high lactate and low oxygen (198). Comparable spatial gradients also exist within TB granulomas, which exhibit unique immune transcripts, proteomic profiles, and diverse cell subsets. These granulomas have locally coordinated immunoregulatory programs with broader systemic impacts that define active TB (44, 67, 69, 199).

Oxygen tension gradients and changes in vasculature linked to granulomas cause limited perfusion and central hypoxia (200, 201). Redox balance within granulomas also plays a critical role (202). Redox-modulating agents such as the thioredoxin reductase inhibitor auranofin (203) and cysteine-reactive inhibitors like ebselen (204, 205) may regulate the redox balance and immune effects in the granuloma microenvironment through distinct mechanisms, thereby exhibiting potential antimicrobial properties. In addition, it’s probable that spatial variations in lactate metabolism and varied interactions among different cell types occur within the context of TB granulomas, given the similarities between tumor and TB microenvironments. These factors might serve as potential HDT targets for TB treatment. Furthermore, Chen et al. (89) have shown that lactate dehydrogenase expression and NBS1 lactylation are associated with clinical resistance to neoadjuvant chemotherapy, resulting in reduced patient survival rates. They also demonstrated that stiripentol, an anti-epileptic drug, significantly reduced lactate production and NBS1 lactylation. In mouse models, stiripentol combined with chemotherapy or radiotherapy showed strong synergistic antitumor effects and prolonged survival time. Related clinical trials have been registered (registration number: ChicTR2400083649). Overall, lactate has demonstrated great potential in treating tuberculosis and lung cancer.

There has been substantial advancement in identifying therapeutic targets that exploit metabolite-sensing signaling pathways to halt cancer progression (70). Therefore, targeting shared metabolic changes in TB and LC, such as those involving arginine, lactate, and glutamine, might provide common therapeutic targets for TB and LC.

In summary, we have provided a comprehensive review of the complex relationship between LC and TB, covering topics from epidemiology to underlying molecular mechanisms. We focus on the latest advancements in spatial multi-omics, as well as developments in immune microenvironments, cellular elasticity, immunometabolism, and microbiomics. Based on these insights, we propose future research directions and potential treatment strategies for TB-LC.
